# Research progress on exosomes in podocyte injury associated with diabetic kidney disease

**DOI:** 10.3389/fendo.2023.1129884

**Published:** 2023-03-20

**Authors:** Jiao Li, Shanshan Zheng, Chaoqun Ma, Xuexun Chen, Xuan Li, Shengjie Li, Ping Wang, Ping Chen, Zunsong Wang, Wenbin Li, Yipeng Liu

**Affiliations:** ^1^ Department of Nephrology, The First Affiliated Hospital of Shandong First Medical University & Shandong Provincial Qianfoshan Hospital, Jinan, China; ^2^ Department of Emergency, Shandong Provincial Hospital Affiliated to Shandong First Medical University, Jinan, China; ^3^ Department of Nephrology, Affiliated Hospital of Weifang Medical University, School of Clinical Medicine, Weifang Medical University, Weifang, China; ^4^ Department of Nephrology, Shandong Provincial Qianfoshan Hospital, Shandong University, Jinan, China; ^5^ Nephrology Research Institute of Shandong Province, Jinan, China

**Keywords:** diabetic kidney disease, podocyte injury, exosomes, microRNA, biomarkers, communication

## Abstract

Diabetic kidney disease (DKD), a common cause of end-stage renal disease, is a serious complication that develops with the progression of chronic diabetes. Its main clinical manifestations are persistent proteinuria and/or a progressive decline in the estimated glomerular filtration rate. Podocytes, terminally differentiated glomerular visceral epithelial cells, constitute the glomerular filtration barrier together with the basement membrane and endothelial cells, and the structural and functional barrier integrity is closely related to proteinuria. In recent years, an increasing number of studies have confirmed that podocyte injury is the central target of the occurrence and development of DKD, and research on exosomes in podocyte injury associated with DKD has also made great progress. The aim of this review is to comprehensively describe the potential diagnostic value of exosomes in podocyte injury associated with DKD, analyze the mechanism by which exosomes realize the communication between podocytes and other types of cells and discuss the possibility of exosomes as targeted therapy drug carriers to provide new targets for and insights into delaying the progression of and treating DKD.

## 1 Introduction

Diabetic kidney disease (DKD), one of the serious conditions included in diabetes mellitus, is a common cause of end-stage renal disease. Additionally, DKD is ranked first among the causes of chronic kidney disease (1, 2), with high morbidity and mortality. Epidemiological data suggest that approximately 40% of diabetic patients could develop DKD (3). The main pathological features of DKD include progressive thickening of the glomerular basement membrane, mesangial matrix expansion, extracellular matrix accumulation, tubulointerstitial fibrosis and podocyte injury (4, 5). At present, the available treatments for DKD include lifestyle interventions such as strict control of blood glucose, blood pressure, blood lipids, restriction of protein intake, and medicine therapy including renin-angiotensin-aldosterone system inhibitors, endothelin-1 receptor antagonists, new hypoglycemic agents, dipeptidyl peptidase 4 inhibitors, sodium-glucose cotransporter 2 inhibitors and glucagon-like peptide-1 receptor agonists, and both of which play a role in reducing proteinuria and cardiovascular mortality. However, the existing treatments can only delay disease progression to a certain extent and fail to effectively prevent or reverse the progression of DKD. Therefore, it is essential to carry out research on the mechanisms of DKD and explore new treatments.

Numerous studies have shown that podocyte injury is one of the main factors in the initial pathogenesis and progression of DKD (6–8), and has become the focus of recent studies. Podocytes, located on the lateral side of the glomerular basement membrane, constitute the glomerular filtration barrier together with the basement membrane as well as endothelial cells with fenestrations, and are the most important cells that perform filtration functions. Podocytes are highly specialized and terminally differentiated cells, and as a result, harmful factors, such as mechanical tension, oxidative stress, and inflammatory stimuli, can cause permanent podocyte damage. During the development of DKD, podocyte apoptosis or detachment from the glomerular basement membrane often leads to a decrease in the number of podocytes, which disrupts the integrity of the glomerular filtration membrane, causes proteinuria, and further accelerates foot process detachment. Animal studies have shown that a loss of more than 20% of podocytes results in irreversible glomerular damage, leading to end-stage renal disease (9). Podocyte phenotypic changes (cell transdifferentiation, hypertrophy, apoptosis, foot process fusion or disappearance), disruption of structural function in the slit diaphragm, and podocyte detachment are the main causes of proteinuria and DKD progression (8–10). Thus, the extent of podocyte injury and loss plays a decisive role in the speed of DKD progression. These studies help us understand the multifactorial nature of podocyte injury and the pathophysiological mechanisms underlying the development of DKD, and further suggest that podocyte therapeutic intervention may be an effective strategy for the prevention and treatment of DKD in the future.

In recent years, exosomes have attracted extensive attention as emerging biomarkers. Exosomes, which are vesicles released by a variety of cell types, crosstalk with adjacent or distant cells under different physiological and pathological conditions. Studies have found that exosomes can improve renal function and histological pathology, inhibit tissue apoptosis, reduce oxidative stress levels, and inhibit inflammatory cell infiltration. Additionally, they can play a role in promoting tumor cell migration. The ability of exosomes to selective package and intercellularly deliver microRNAs (miRNAs) has attracted widespread interest. These functions are involved in DKD pathogenesis, which suggests that exosomes could be biomarkers of DKD and potentially valuable as a new treatment strategy (11, 12).

## 2 Biological characteristics of exosomes

Exosomes are a class of nanoscale extracellular small vesicles surrounded by lipid bilayers with diameters of approximately 30-100 nm that are released into the external environment by cells under different physiological or stress conditions and contain abundant contents. Approximately 30 years ago, exosomes secreted by sheep reticulocytes were first identified and initially thought to be “garbage trucks” that remove cellular metabolites (13). However, after the discovery that exosomes carry mRNA and miRNA in 2006, they were reconsidered based on this new perspective. As a novel carrier of genetic information, exosomes play an important role in cell-to-cell communication. Formed mainly in the endocytic pathway with plasma membrane invagination, exosomes undergo early endosomal, late endosomal and content loading processes to further form mature multivesicular bodies. These mature multivesicular bodies either fuse with lysosomes, whose contents are degraded, or they are released into the extracellular space after fusion with the plasma membrane and taken up by recipient cells to induce cell−cell crosstalk through a variety of mechanisms, including endocytosis, pinocytosis, phagocytosis, and membrane fusion. Mature multivesicular bodies can also directly activate cell surface receptors and specific signaling pathways *via* ligands or presenting antigens (14, 15). Moreover, most exosomes have an evolutionarily conserved set of marker molecules, including tetrapeptides (CD81, CD63, and CD9), Alix, and Tsg101, but they also have unique tissue- or cell-type-specific proteins that reflect their cellular origin. As messengers of intercellular communication, they can be secreted by almost all types of cells in the human body and are widely distributed in various body fluids (e.g., plasma, urine, saliva, cerebrospinal fluid, breast milk, amniotic fluid, semen, thoracic ascites, etc.) and abnormal tissues (e.g., tumor tissue), carrying a variety of bioactive substances, such as multiple proteins/peptides, nucleic acids (DNA, mRNA, miRNA, other noncoding RNAs) and lipids. Exosomes are widely involved in multiple biological regulatory processes, including cell communication, migration, angiogenesis, inflammatory response, and tumor growth (16).

## 3 Exosomes associated with podocyte injury as potential biomarkers for the diagnosis of DKD

The main clinical manifestations of DKD are persistent albuminuria and/or a reduced estimated glomerular filtration rate (eGFR). The urine albumin creatine ratio has been widely used as a conventional biomarker for the onset of DKD and its progression to end-stage renal disease, but there are some differences between the clinical manifestations and histopathological damage of DKD. In recent years, it has been found that renal injury, or even deterioration, may also be present in normal or slightly reduced renal function or in the absence of proteinuria (17, 18), and there is conflicting evidence for the specificity and sensitivity of biomarkers for proteinuria. Therefore, it is urgent to find better biomarkers for the diagnosis of DKD. In 2011, a study by Zheng et al. (19) showed that the mRNA expression of urinary podocyte injury markers (synaptopodin, podocalyxin, CD2-AP, α-actin4 and podocin) was significantly increased, and negatively correlated with eGFR, in DKD patients compared with those of healthy controls. This finding shows that there is elevated podocyte excretion in DKD patients, and that the quantification of podocyte-related molecules in urine reflects the severity of proteinuria and renal damage, suggesting that these podocyte-specific genes may serve as biomarkers for the progression of DKD. In 2014, Lv et al. (20) reported that podocyte-associated CD2AP mRNA expression in urinary exosomes correlated with renal function, proteinuria levels, and severity of renal fibrosis, supporting the critical role of podocyte loss in the development of proteinuria. As a novel noninvasive tool, urinary exosome mRNA expression of CD2AP enables the assessment of renal function and fibrosis in patients with kidney disease. In a 2018 clinical study, Abe et al. (21) found that although proteinuria levels were approximately the same in both patient groups, DKD patients showed a significant excretion of WT1 exosomes in their urine compared to patients with minimal change nephrosis and further observed a rapid decrease in eGFR in DKD patients with high levels of exosomal WT1 mRNA expression. In addition, exosomes extracted from the culture medium of high-glucose-treated podocytes showed high expression of WT1 mRNA *in vitro*. Therefore, podocyte-associated exosome WT1 mRNA monitoring in urine is a potential biomarker for the identification of DKD and may be superior to proteinuria biomarker detection. In 2019, Sakurai et al. (22) proposed that quantification of Elf3 levels in urinary exosomes may help to assess podocyte injury due to activation of transforming growth factor-β (TGF-β) signaling, which could be used as a new predictive marker for early diagnosis of DKD. In recent years, the diagnostic value of different podocyte injury-associated exosomal contents as biomarkers for DKD has received increasing attention. Clinical data (23) found that urinary exosomal miRNA-22 was negatively correlated with nephrin protein levels, which reflects the status of podocyte damage and positively correlated with Mogensen stage in DKD patients, which reflects the severity of the disease. Under high glucose conditions, the expression of miR-221 (24) and let-7f-2-3p (25) in damaged podocyte-derived exosomes was significantly upregulated, and the expression of miR-1981-3p, miR-3473, miR-7224-3p and miR-6538 (25) was significantly downregulated, indicating that these exosome-derived miRNAs have suitable diagnostic potential for DKD. By studying the proteomics of urinary exosomes in healthy controls and DKD patients, Zubiri et al. (26) found that AMBP and MLL3, which were differentially expressed, also showed favorable potential as diagnostic markers for DKD.

## 4 Exosomes mediate communication between other cells and podocytes

**Figure 1:**
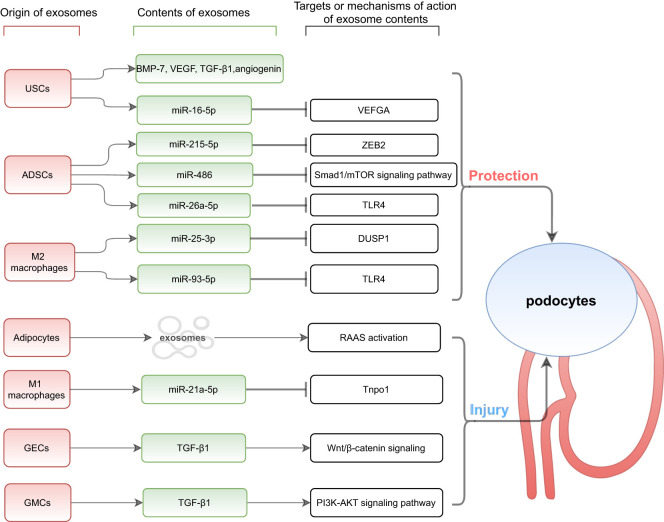
Exosomes mediate communication between other cells and podocytes. USCs, urine-derived stem cells; ADSCs, adipose-derived stem cells; GECs, glomerular endothelial cells; GMCs, glomerular mesangial cells; BMP-7, bone morphogenetic protein-7; VEGF, vascular endothelial growth factor; TGF-β 1, transforming growth factor-β 1; VEFGA, vascular endothelial growth factor A; ZEB2, zinc finger E-box-binding homeobox-2; TLR4, Toll-like receptor 4; DUSP1, dual specificity protein phosphatase1; RAAS, renin-angiotensin-aldosterone system; Tnpo1, transportin 1.

It was found that the number and contents of exosomes secreted by damaged renal cells are altered under conditions of hypoxia, acidic pH, toxins, high glucose and oxidative stress (27). Exosomes secreted by inflammatory cells can induce the release of inflammatory cytokines and promote the accumulation of inflammatory cells; exosomes secreted by damaged renal cells can be transferred to other normal renal cells, inducing cell-to-cell interactions (15) to change their phenotype. As a result of their ability to mediate communication between different cells, exosomes and their role in podocyte injury in DKD has been largely investigated (**Figure 1**).

As an important posttranscriptional regulator of gene expression, miRNAs are short noncoding RNAs of 18-22 nucleotides in length that bind to the 3’ untranslated region (3’ UTR) of mRNAs and regulate cell growth, differentiation, apoptosis, and proliferation by inducing mRNA degradation or inhibiting translation to interfere with protein synthesis (28). Compared with free miRNAs, exosomal miRNAs are stable and protected by the exosomal plasma membrane from degradation by RNases in biological fluids or the external environment. Lv et al. (29) found that exosomal miRNAs could be detected even when kept at 4°C for 24 hours, stored at 80°C for 12 months, or repeatedly frozen and thawed five times. Several studies have shown that miRNA dysregulation exists in most renal disease processes and is closely related to autophagy, fibrosis, inflammation, epithelial-mesenchymal transition (EMT) and other mechanisms (30–32), therefore exosomal miRNAs are gradually becoming a popular topic for studies related to podocyte injury in DKD.

### 4.1 Urine-derived stem cell exosomes and podocyte injury

Urine-derived stem cells are a type of stem cell that are found in urine and have characteristics similar to mesenchymal stem cells. They have a strong potential for self-proliferation, multidirectional differentiation, and secretion of bioactive substances (33). Urine-derived stem cells have been shown to mediate the repair of podocyte injury by secreting exosomes and can express the podocyte marker CD146 (34). Researchers have further demonstrated the possibility that urine-derived stem cells are homologous to podocytes by locating the origin of urine-derived stem cells more precisely in the renal utricle parietal epithelial cells or podocytes based on the identification of cell-specific markers (35). Jiang et al. (36) found that exosomes from urine-derived stem cells reduced high-glucose-induced apoptosis of podocytes in an *in vitro* cell model study of the effects of high glucose medium on podocyte injury. Further studies showed that urine-derived stem cell exosomes contained growth factors such as TGF-β1, angiopoietin, and bone morphogenetic protein-7 that may be related to angiogenesis and cell survival. Similarly, studies in streptozotocin-induced rat models showed that intravenous injection of exosomes derived from normal human urine-derived stem cells can reduce urine volume and urinary microalbumin excretion, inhibit caspase-3 overexpression in podocytes and tubular epithelial cells and prevent podocyte and tubular epithelial cell apoptosis in diabetic rats. Moreover, Duan et al. (37) screened miR-16-5p from a bioinformatics database and found that it could bind and regulate vascular endothelial growth factor A. High glucose stimulation inhibited miR-16-5p and nephrin expression in podocytes and promoted vascular endothelial growth factor A expression. *In vitro* coculture assays further demonstrated that the transfer of miR-16-5p from urine-derived stem cells to podocytes was dependent on exosomes. In addition, miR-16-5p effectively inhibited the expression of vascular endothelial growth factor A in high-glucose-stimulated podocytes and alleviated podocyte injury induced by high glucose. Similarly, animal experimental models of diabetic rats showed that significantly increased expression of miR-16-5p in urine-derived stem cell exosomes reduced the degree of podocyte injury, improved podocyte morphology, cell number, foot process width, mesangial area, and inflammatory factor levels and inhibited podocyte apoptosis. In conclusion, urine-derived stem cell exosomes overexpressing miR-16-5p provide a novel strategy for preventing podocyte injury and inhibiting angiogenesis. In addition to noninvasive and simple extraction methods, urine-derived stem cells have a natural advantage over other mesenchymal stem cells because they exist in the urinary system and are mostly derived from kidney tissue. Overall, urine-derived stem cell-derived exosomes are important for the prevention and treatment of DKD.

### 4.2 Adipose-derived exosomes and podocyte injury

As a major site for storing excess energy, adipose tissue is not only a major source of stored energy, but also an important part of the endocrine system that has paracrine and autocrine functions, including synthesizing and secreting many biologically active substances that regulate metabolic homeostasis. In obesity-related inflammatory conditions, adipose tissue can release tumor necrosis factor-α, interleukin-6, and retinol-binding protein-4, contributing to the development of systemic insulin resistance (38). Zhang et al. (39) found that exosomes secreted by adipocytes could serve as mediators in communication between adipose tissue and other metabolic organs, regulating blood glucose homeostasis. An *in vitro* study (40) found that podocytes showed activation of the renin-angiotensin-aldosterone system and decreased expression of podocyte-specific proteins (nephrin and podocin) when exosomes extracted from subcutaneous fat of mice on a high-fat diet were co-cultured with podocytes. It is suggested that adipose-derived exosomes may promote activation of the renin-angiotensin-aldosterone system in the kidney and mediate podocyte injury. Moreover, adipose-derived mesenchymal stem cells are adult stem cells derived from adipose tissue. In recent years, adipose-derived mesenchymal stem cell exosomes have shown promising results in the treatment of DKD. Jin et al. (41) demonstrated that these exosomes mediated miR-215-5p shuttling to podocytes, inhibited podocyte EMT by suppressing the transcription of ZEB2, and ameliorated podocyte dysfunction. ZEB2 is a DNA-binding transcription factor that plays an important role during EMT, migration, and invasion (42). Mechanistically, adipose-derived mesenchymal stem cell exosomes inhibit ZEB2 transcription through miR-215-5p, which could serve as a protective strategy from podocyte EMT. A previous study by Jin et al. (4) also confirmed that miR-486 carried by exosomes from adipose-derived mesenchymal stem cells could act as an activator of autophagy to alleviate high-glucose-induced podocyte injury in mice. Adipose-derived mesenchymal stem cell-derived exosomes were also able to reverse the low expression of miR-486 in podocytes of DKD mice. MiR-486 targeted and reduced Smad1 expression, which in turn inhibited mTOR activation, resulting in increased autophagy, reduced podocyte apoptosis and improved podocyte injury. In addition, Duan et al. (43) demonstrated that exosomes from adipose-derived mesenchymal stem cells improved DKD symptoms in diabetic mice, as shown by a decrease in proteinuria, serum creatinine, and urea nitrogen. Adipose-derived mesenchymal stem cell-derived exosomes mediated the transfer of miR-26a-5p to high-glucose-induced mouse podocytes to protect podocytes from injury, enhance their survival and inhibit podocyte apoptosis (characterized by decreased Bax, caspase3 breakage, and increased Bcl-2) by targeting TLR4, inactivating the nuclear factor-kB pathway and downregulating vascular endothelial growth factor A. Previous studies have shown that adipose-derived mesenchymal stem cell transplantation also attenuates podocyte injury in DKD by activating klotho expression and inhibiting the Wnt/β-catenin signaling pathway (44). Overall, adipose-derived mesenchymal stem cell transplantation holds promise as a potential treatment for DKD (45).

### 4.3 Macrophage-derived exosomes and podocyte injury

Macrophages are not only phagocytic intrinsic immune cells but also important mediators of tissue homeostasis and host defense. Numerous studies have shown that monocyte-macrophage infiltration is an important pathological feature in the development of DKD (46). M1 macrophages promote the progression of DKD by secreting proinflammatory factors (IL-1β, IL-6, IL-12, IL-23, and TNF-α); M2 macrophages inhibit the development of DKD by secreting anti-inflammatory factors (IL-10 and TGF-β) (47, 48). The balance of M1/M2 macrophage polarized phenotypes determines the fate of organs in inflammation or injury. In a study by LAN et al. (49), miR-25-3p was found to be highly expressed in exosomes derived from M2 macrophages in the tumor microenvironment and functioned to promote cancer cell migration and invasion. HUANG et al. (50) further found that M2 macrophage-derived exosome miR-25-3p activated podocyte autophagy and attenuated podocyte apoptosis and EMT by inhibiting DUSP1 expression, thereby protecting podocytes from high-glucose-induced injury. The experiment conducted by ZHUANG et al. (51) to investigate the effect of γ-aminobutyric acid in high-glucose-induced podocyte injury showed that M1 macrophages aggravated high-glucose-induced podocyte injury by secreting exosomes to reduce the proliferation ability and increase the apoptosis rate of cells under high glucose conditions. In the study, miR-21a-5p expression was found to be significantly upregulated in macrophage-derived exosomes, and miR-25-3p expression showed no significant alteration. Tnpo1/ATXN3 was demonstrated to be a target of miR-21a-5p/miR-25-3p by a series of experiments, including online database prediction, luciferase analysis and functional validation. Finally, functional complementation experiments demonstrated that γ-aminobutyric acid significantly reduced the podocyte apoptosis rate and attenuated podocyte injury by reversing the M1/M2 polarization of macrophages from proinflammatory to anti-inflammatory under high glucose conditions and regulating the miR-21a-5p-Tnpo1/miR-25-3p-ATXN3 signaling axis of macrophage-derived exosomes. In the same year, WANG et al. (52) similarly demonstrated that M2 macrophage-derived exosomes could exert nephroprotective effects by regulating the miR-93-5p/TLR4 axis to inhibit lipopolysaccharide-induced podocyte apoptosis *in vitro*. The above studies confirm the importance of paracrine communication *via* exosomes between macrophages and podocytes, and provides a novel drug target for the treatment of DKD patients.

### 4.4 Glomerular endothelial cell/mesangial cell-derived exosomes and podocyte injury

As the first barrier of the glomerular filtration membrane, glomerular endothelial cells are more vulnerable to damage from elevated blood glucose and lipid levels and inflammatory factors due to direct contact with circulating substances in the blood. Moreover, a continuous hyperglycemic environment generates large amounts of reactive oxygen species, which promote the development of DKD. Wu et al. (53) found that under continuous high glucose stimulation, glomerular endothelial cells undergo mesenchymal transition with a significant increase in the number of secreted exosomes that trigger EMT and barrier dysfunction of podocytes by transferring mRNA of TGF-β1, a potent profibrotic cytokine. It has been shown to be a potent trigger of podocyte EMT, mediating podocyte injury and proteinuria formation through typical Wnt/β-catenin signaling. Further *in vivo* studies revealed that exosome secretion by glomerular endothelial cells stimulated by high glucose could activate glomerular mesangial cells, leading to mesangial cell proliferation and extracellular matrix deposition in the mouse mesangial area.

Glomerular mesangial cells are involved in multiple pathophysiological/injury pathways in the diabetic state. An *in vitro* study by Wang et al. (54) found that high-glucose-induced release of exosomes from glomerular mesangial cells mediated crosstalk between glomerular mesangial cells and podocytes through activation of the PI3K-AKT signaling pathway in podocytes by TGF-β1, which in turn induced podocyte apoptosis and inhibited podocyte adhesion. Based on this study, it was found that berberine treatment could reduce podocyte injury by downregulating TGF-β1 receptor expression, which reduced apoptosis and increased podocyte adhesion ability and podocyte-specific marker protein (nephrin, podocin and WT-1) expression. Berberine treatment exerts a protective effect on the process of podocyte injury induced by high-glucose-induced exosomes that are from glomerular mesangial cells, which provides a potential therapeutic agent for DKD.

## 5 Prospect

Many therapies for the treatment of DKD are currently being developed, including stem cell therapies and targeted agents that influence pathways such as inflammation, oxidants, or pro-fibrosis activation during the progression of DKD. The therapeutic potential of stem cells is derived in part from their secretory capacity, in which exosome secretion plays a particularly important role. Exosomes not only partially express the therapeutic activity of parental cells but also have the autonomous ability to target specific diseased tissues. SUN et al. (55) found that exosomes can successfully carry and deliver the anti-inflammatory molecule curcumin. The formation of exosome-curcumin complexes greatly increased the solubility, *in vitro* stability, bioavailability and anti-inflammatory activity of curcumin. Wang et al. (56) also developed a hydrogel with bioactive substances from exosomes that accelerated the healing process of diabetic wounds by enhancing neovascularization and promoting cell proliferation. In addition, it has been confirmed that human bone marrow stem cells (57), umbilical cord mesenchymal stem cells (58), and adipose-derived mesenchymal stem cells (59) can differentiate into insulin-secreting cells. By injecting the stem cells into diabetic patients, we used the biological characteristics of exosomes to restart the body’s own repair and regeneration ability, including its ability to continuously repair damaged islet tissue and replace damaged pancreatic β cells in the body, restore islet function, and promote insulin secretion to effectively treat diabetes and its complications. With the continuous improvement and progress of technology, artificially collecting or preparing stem cell-derived exosomes containing many beneficial components, such as miR-16-5p, miR-215-5p, miR-486, miR-26a-5p, miR-25-3p, miR-93-5p, etc., or preparing exosomes containing inhibitors of identified harmful components, such as miR-21a-5p, TGF-β, etc., for the clinical treatment of podocyte injury is expected to be a major breakthrough in the field of DKD prevention. With the continuous increase in knowledge concerning exosomes in the study of DKD mechanisms, it is believed that exosomes and their contents will become potential targets for clinical intervention of DKD.

In summary, this review discusses the diagnosis, pathophysiological mechanisms and therapeutic application prospects of exosomes in podocyte injury associated with DKD. As an endogenous drug nanodelivery system with unique advantages, such as innate stability, low immunogenicity, high delivery efficiency and targeted “homing” ability, exosome therapy is currently the leading candidate in the development of new therapeutic approaches to target drug carriers for DKD treatment. Exosomes have broad prospects for clinical application and are worthy of in-depth exploration and research.

## Author contributions

YL and WL contributed to conception and design of the study. JL wrote the first draft of the manuscript. SZ, SL, PW, PC and ZW revised the manuscript. CM, XC and XL reviewed this article. All authors contributed to the article and approved the submitted version.
